# Prospective Study on CAD/CAM Nano-Ceramic (Composite) Restorations in the Treatment of Severe Tooth Wear

**DOI:** 10.3290/j.jad.b2838137

**Published:** 2022-03-24

**Authors:** Luuk A.M.J. Crins, Niek J.M. Opdam, Cees M. Kreulen, Bernadette A.M.M. Sterenborg, Ewald M. Bronkhorst, Wietska A. Fokkinga, Marie-Charlotte D.N.J.M. Huysmans, Bas A.C. Loomans

**Affiliations:** a Lecturer and Dentist, Radboud University Medical Center, Radboud Institute for Health Sciences, Department of Dentistry, Nijmegen, The Netherlands. Data acquisition and interpretation, performed statistical analyses, drafted and critically revised the manuscript, gave final approval and agreed to be accountable for all aspects of the work.; b Assistant Professor, Radboud University Medical Center, Radboud Institute for Health Sciences, Department of Dentistry, Nijmegen, The Netherlands. Conception and design, treatment procedures, data interpretation, critically revised the manuscript, gave final approval and agreed to be accountable for all aspects of the work.; c Assistant Professor, Radboud University Medical Center, Radboud Institute for Health Sciences, Department of Dentistry, Nijmegen, The Netherlands. Design, data interpretation, performed all statistical analyses, critically revised the manuscript, gave final approval and agreed to be accountable for all aspects of the work.; d Dentist, Radboud University Medical Center, Radboud Institute for Health Sciences, Department of Dentistry, Nijmegen, The Netherlands. Conception and design, treatment procedures, critically revised the manuscript, gave final approval and agreed to be accountable for all aspects of the work.; e Biostatistician, Radboud University Medical Center, Radboud Institute for Health Sciences, Department of Dentistry, Nijmegen, The Netherlands. Data acquisition, critically revised the manuscript, gave final approval and agreed to be accountable for all aspects of the work.; f Assistant Professor, Radboud University Medical Center, Radboud Institute for Health Sciences, Department of Dentistry, Nijmegen, The Netherlands. Treatment procedures, critically revised the manuscript, gave final approval and agreed to be accountable for all aspects of the work.; g Professor, Radboud University Medical Center, Radboud Institute for Health Sciences, Department of Dentistry, Nijmegen, The Netherlands. Contributed to conception, design, data interpretation, critically revised the manuscript, gave final approval and agreed to be accountable for all aspects of the work.; h Professor, Radboud University Medical Center, Radboud Institute for Health Sciences, Department of Dentistry, Nijmegen, The Netherlands. Project leader of the Radboud Tooth Wear Project, contributed to conception, design, enrollment of patients, data acquisition and interpretation, critically revised the manuscript, gave final approval and agreed to be accountable for all aspects of the work.

**Keywords:** tooth wear, composite resins, occlusal vertical dimension, dental restoration, oral health-related quality of life, CAD/CAM, oral rehabilitation, restorative materials, minimally invasive

## Abstract

**Purpose:**

The aim of this prospective study was to evaluate the clinical performance of minimally invasive, CAD/CAM nano-ceramic (composite) restorations in patients with severe tooth wear, the effect of the restorative treatment on the oral health-related quality of life (OHRQoL), and the etiology of tooth wear as a risk factor for restoration failure.

**Materials and Methods:**

Patients with generalized severe tooth wear were included. Restorations (LAVA Ultimate, 3M Oral Care) were cemented (RelyX Ultimate, 3M Oral Care) on all teeth and were evaluated after 1 month and 1 year. OHRQoL was assessed via questionnaires at baseline and after 1 year. Differences were evaluated (paired t-test). Two mechanical tooth-wear lesions resulting from tooth-tooth contact, and 3 chemical tooth wear lesions resulting from intrinsic or extrinsic acids dissolving natural hard tooth substance, were evaluated to assess the etiology of tooth wear in association with restoration failure using multilevel logistic regression analyses (p < 0.05).

**Results:**

Twenty-one patients (age: 41.7 ± 10.4 years) were evaluated after 1 year (13.5 ± 1.2 months). 568 indirect CAD/CAM restorations were placed. None were replaced or lost. Twelve were repaired and 10 were refurbished. Success rates were 100% to 97.2%. Questionnaires showed a significant positive impact of the treatment on OHRQoL (p < 0.001). The presence of mechanical lesions did not pose a higher risk for restoration failure (p = 0.78). The presence of chemical lesions showed a lower risk of restoration failure (p = 0.002).

**Conclusion:**

The use of minimally invasive, CAD/CAM nano-ceramic (composite) restorations in the restorative treatment of severely worn dentitions showed satisfactory results in the short term.

Tooth wear is a physiological process that may become pathological, resulting in pain, masticatory dysfunction, severely impaired esthetics, and loss of oral health-related quality of life (OHRQoL).^19,33^ Restorative intervention is then a treatment strategy to regain function of the dentition^19^ and to improve the OHRQoL.^33^ Until now, there has been no evidence regarding the best restorative intervention or material for severely worn dentitions.^23^

The most commonly reported restorative treatment for worn dentitions is the placement of directly applied light-cured composite restorations on all teeth. This technique is particularly minimally invasive, as no or very restricted preparation of tooth tissue is required. Favorable Annual Failure Rates (AFR) of about 2%-3% using these restorations in load-bearing areas have been reported.^3,11,20,24^ Less favorable AFRs (16% and higher) have also been reported, indicating differences in success.^5,14^ In patient-centered care, the outcome of restorative rehabilitation should also be evaluated from the perspective of OHRQoL, apart from the evaluation of restoration and material performance. The Oral Health Impact Profile (OHIP),^32^ comprising specific subjective questions on diverse domains, and the Orofacial Esthetic Scale (OES),^15^ focusing on the esthetic domain, have been used to assess the improvement of OHRQoL after restorative treatment of severe tooth wear,^16,33^ with positive results.

Reconstruction of the worn dentition is complicated, as the procedure requires an increase of vertical dimension of occlusion (VDO). Although specific techniques^1,25,27,31^ can be used to help operators to shape directly applied restorations, it remains complex.

This problem is avoided entirely when using indirect techniques, facilitating both shaping of restorations and obtaining the desired VDO. These techniques range from conventional impressions, which are used to construct restorations in a dental laboratory,^28^ to a complete digital workflow.^10^ With developments in adhesive dentistry over the years, a shift in indirect techniques has occurred from subtractive, invasive procedures with circumferential preparations^8^ and (possibly) crownlengthening procedures^22^ towards additive and conservative procedures,^10,21^ which are recommended in a European consensus statement.^19^

Non-retentive indirect restorations on all teeth to restore worn dentitions have been described. Both lithium-disilicate glass-ceramic restorations in a moderately invasive procedure,^7^ and CAD/CAM polymer-infiltrated ceramic network (PICN) restorations in a non-invasive (non-prep) procedure,^21,26^ showed good outcomes in the mid- and short term, respectively. This suggests a place for additive indirect techniques in the restorative treatment of tooth wear, comprising minimally invasive CAD/CAM techniques. Composite and especially PICN materials present advantages for the conservative management of tooth wear, as they offer strong adhesive bonds and good repairability.^6,13^ CAD/CAM techniques also offer superior materials properties, such as lack of voids and a higher degree of polymerization.^2,30^

Severe tooth wear patients may constitute a high-risk population for restorative care. The etiological factors of tooth wear are likely still present after restorative treatment has been conducted, possibly impairing the longevity of the restorations.^11^ Particularly in patients with mainly mechanical tooth wear, more fractures of the restorative treatment are expected. Distinguishing between patients with mechanical and chemical tooth wear is difficult, as the phenomenon of tooth wear is always multifactorial and scientific evidence is limited.^12^

In the Radboud Tooth Wear Project, a group of patients with severe tooth wear was restoratively treated with minimally invasive CAD/CAM nano-ceramic (composite) restorations, as described in a case report (Kreulen et al, accepted by J Adhes Dent 2022). The aim of this study was to evaluate the clinical performance of these restorations, to evaluate the effect of the restorative treatment on OHRQoL, and to evaluate the etiology of tooth wear as a risk factor for restoration failure.

## Materials and Methods

### Design

This was a prospective clinical study evaluating the rehabilitation of tooth wear using minimally invasive CAD/CAM nano-ceramic composite restorations (LAVA Ultimate, resin nano-ceramic composite, 3M Oral Care; St Paul, MN, USA). The local medical ethics committee (CMO Arnhem-Nijmegen) confirmed that their approval was not required for this study (file nr. 2014-1252). Prior to commencement of the study, it was registered on ClinicalTrials.Gov (NCT02957734).

### Patient Selection

Patients with severe tooth wear were referred by their general dental practitioner to the RTWP (Radboud Tooth Wear Project) at the Department of Dentistry of the Radboud university medical center in Nijmegen (The Netherlands). Inclusion was based on the following criteria: 1) patients age ≥ 18 years; 2) moderate to severe generalized tooth wear with functional problems and demand for treatment; 3) full dental arches, although one diastema in the posterior area was allowed; 4) required increase of VDO on first molars of at least 3 mm.

Exclusion criteria were 1) (history of) temporomandibular dysfunction; 2) advanced periodontitis; 3) deep caries lesions; 4) multiple endodontic problems; 5) local or systemic conditions that would contra-indicate dental procedures. Patients with specific individual risk factors, such as gastroesophageal reflux disease or parafunctional habits as grinding/clenching, were not excluded. All patients signed an informed consent document before entering the study.

### Baseline Registration

At baseline, patients were asked to complete the OHIP-NL questionnaire^17,32,36^ and the OES-NL questionnaire.^15^ Patients completed both questionnaires after instruction. For each statement of the OHIP-NL questionnaire, patients were asked how frequently they experienced the impact of the specific statement. Higher scores imply a lower OHRQoL. Answers were scored on a 5-point ordinal scale, ranging from never (0), hardly ever (1), occasionally (2), and fairly often (3), to very often (4). Three questions, exclusively referring to dentures (no. 9, 18, 39), were omitted from the questionnaire.

The OES-NL questionnaire used an 11-point ordinal scale, ranging from very dissatisfied (0) to very satisfied (10). The questionnaire consisted of 8 items. Items 1 to 7 addressed the appearance of the face, profile, mouth, tooth alignment, tooth shape, tooth color, and gums. The last item (no. 8) characterizes the patient’s overall assessment of orofacial esthetics.

In addition, intra-oral examination, bitewing radiographs, a panoramic radiograph, and intra-oral photographs were collected for treatment planning and documentation. Intra-oral 3D scans (TrueDef, 3M Oral Care) of the dentitions were made in maximum intercuspation. Deficient pre-existing composite restorations and all amalgam restorations were replaced using Filtek Supreme XTE (3M Oral Care) and Scotchbond Universal (3M Oral Care). The restorative status of abutment teeth at baseline, such as endodontic treatment and pre-existing restorations, were registered on the tooth level.

Clinical presentation of tooth wear at baseline was scored using 3D scans and intraoral images by one researcher (LC) on the patient level using an individualized index. This index was based on already existing indices^12,37^ and focused on 5 morphological features of tooth wear lesions.

The features of mechanical tooth wear include a similar degree of wear in all occluding sextants,^12^ and the imprint of mandibular anterior teeth on palatal surfaces of maxillary anterior teeth. The features of chemical tooth wear include the presence of “raised restorations”;^12^ loss of convexities on the palatal surface of maxillary teeth;^12^ and a preserved enamel “cuff” in the gingival palatal crevice of maxillary anterior teeth.^12^

Patients received a score for both mechanical wear etiology – 0 to 2 depending on the numbers of features presented – and chemical wear etiology, with scores from 0 to 3.

### Restorative Procedure

Patients were assigned to one of four operators who were experienced and trained in adhesive dentistry and the specific protocol for placing indirect CAD/CAM restorations. To calibrate clinical procedures, multiple sessions were held before the onset of the study. Furthermore, pilot studies were performed to optimize the restorative protocol.

### Increase of VDO

**Fig 1:**
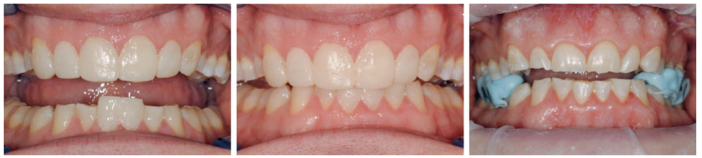
Mockups on the maxillary and mandibular anterior teeth and silicon stops in situ to preserve the new VDO.

The increase of VDO was based on the space needed to lengthen the maxillary and mandibular anterior teeth and on the required interocclusal space of > 1.5 mm to accomodate restoration thickness in load-bearing areas. The estimated new VDO was determined intraorally by applying a free-hand composite mockup on mandibular central incisors (Fig 1). A composite jig was made on the palatal surfaces of the maxillary central incisors with the patient closing along the retruded path of closure,^34^ while there was an occlusal space between the (pre)molars. This new VDO was preserved by adding fast-setting, stiff polyvinylsiloxane silicon bite blocks (Star VPS, Danville Materials; San Ramon, CA, USA) in the right and left posterior areas while the patient was in centric relation^34^ closing on the anterior mockups. Intraoral mock-ups on both maxillary anterior teeth and mandibular central incisors using direct composite were made and used as a jig in the desired increased VDO. With the jig in situ, intraoral posterior stops (Star VPS, Danville Materials) were made in centric relation, using guided closure. The mock-ups were documented using photographs and a 3D scan after the patient gave approval regarding the esthetic aspects.

### Minimally Invasive Preparation

**Fig 2:**
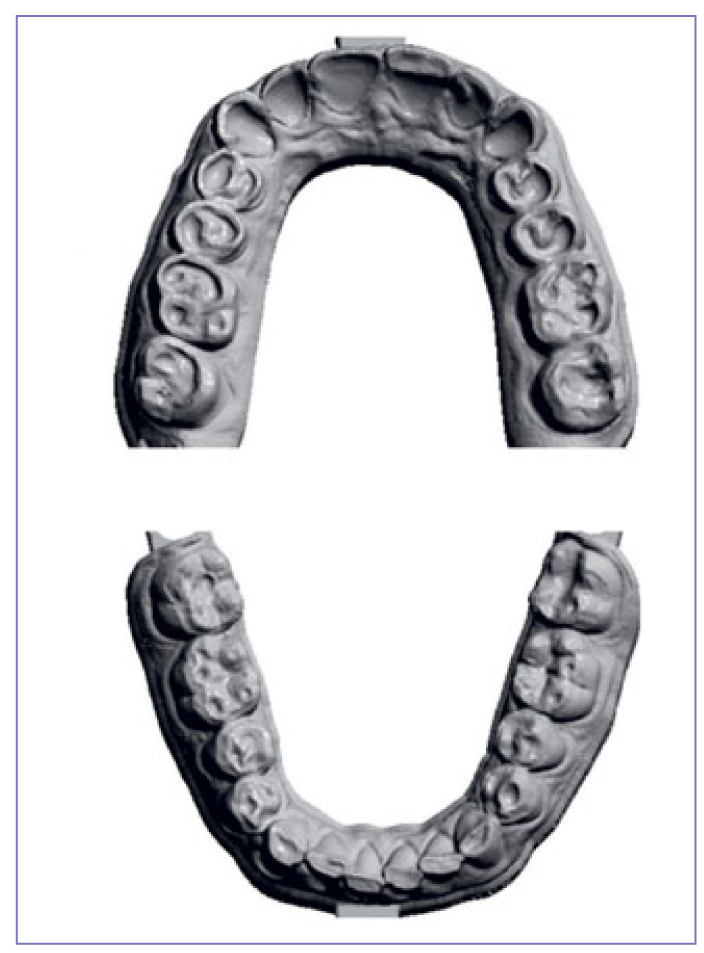
3D scan after preparation.

A minimally invasive tooth preparation was performed. It comprised removal of sharp edges and a shallow chamfer to determine the outline for the dental technician (Fig 2). In selected cases, and especially in lingual surfaces of anterior teeth, seats were prepared where otherwise no “resistance”^34^ of restorations could be obtained and seating of restorations could be enhanced. Intraoral 3D-scans (True Def IOS, 3M Oral Care) were made, including bite registration in the increased vertical dimension, while intraoral stops were placed in situ to mimic the desired, predetermined VDO.

Virtual wax-ups on digital models were made by a dental technician (Elysee Dental, Modern Dental Group; Alphen aan de Rijn, The Netherlands). After agreement of the operator, restorations were milled and finished. To avoid errors of seating during cementation, two series of planning, milling, and cementation of restorations were conducted. The first series was completed for all maxillary and mandibular anterior teeth (palatal/lingual veneer restorations), all second premolars, and all second molars. The second series was completed for all first premolars and first molars. No temporary restorations were placed before placement of the restorations, nor were they placed between the two cementation sessions.

### Cementation of CAD/CAM Nano-Ceramic (Composite) Restorations

At the discretion of the operator, rubber-dam or cotton rolls and suction devices were applied for moisture control. Teeth were cleaned using pumice and then rinsed. LAVA Ultimate restorations were checked for their seating, and then cleaned and roughened using air abrasion (30 µm, CoJet, 3M Oral Care). Both a silane layer (ESPESIL, 3M Oral Care) and an adhesive layer (Scotchbond Universal, 3M Oral Care) were applied to the bonding surface of the restoration. If a pre-existing restoration was present in the abutment tooth, it was air abraded (CoJet). The tooth was etched for 15 s using 37% phosphoric acid (3M Oral Care) and rinsed for 10 s. Silane was applied in case of a pre-existing restoration on the adhesive surface. Next, the adhesive layer was applied on the tooth surface for 20 s and polymerized for 15 s. The restorations were cemented (RelyX Ultimate, 3M Oral Care) and, after removal of cement excess, light cured for 20 s per restoration surface (Bluephase 16i, Ivoclar Vivadent; maximum output 1600 mW/cm^2^). Occlusion and articulation were checked to ensure supported occlusion. Occasionally, small adjustments were made using fine-grit diamond burs and polishing rubbers.

### Direct Veneers

**Fig 3:**
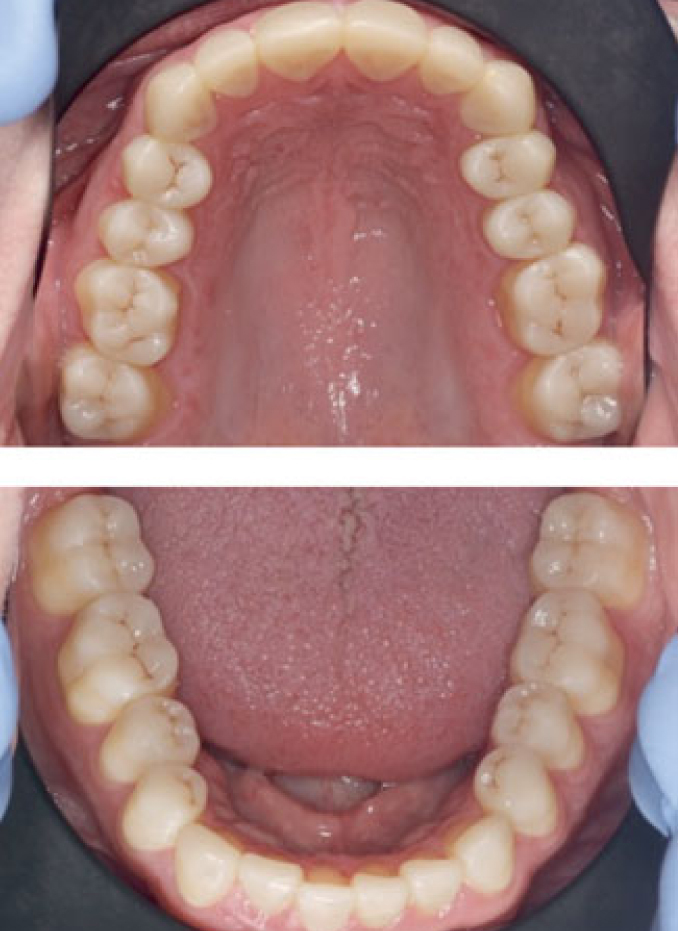
Maxillary and mandibular arches after placement of CAD/CAM composite restorations and facial veneer restorations.

Facial veneers were made using directly applied composite (Filtek Supreme XTE, 3M Oral Care) on all maxillary anterior teeth. In cases where LAVA Ultimate restorations were placed on lingual sides of mandibular anterior teeth, additional direct facial veneers were made (Fig 3). To ensure optimal adhesion between lingual/palatal indirect veneers and the facial veneers, the former were roughened with a bur and air abraded, and silane was used as described above. The facial veneer was considered and evaluated as a separate restoration on the same tooth.

### Follow-up

Recall appointments were scheduled at 1 month and 1 year after treatment. Restorations were assessed (LC, BS, BL) using intraoral examination, intraoral photographs, and 3D scans (TrueDef, 3M Oral Care), focusing on functional (debonding or fracture), biological (caries, endodontic treatment), and esthetic criteria. Restorations with discoloration or roughness needing refurbishment by polishing were not considered failures. Three levels of failure were distinguished:

- restorations with severe deficiencies that were replaced, or in case of an extracted abutment tooth;
- restorations with localized deficiencies that were repaired, or when a completely debonded restoration could be recemented (Fig 4a);
- restorations with small material chippings that received either refurbishment by polishing or needed no intervention (Fig 4b).

**Fig 4a:**
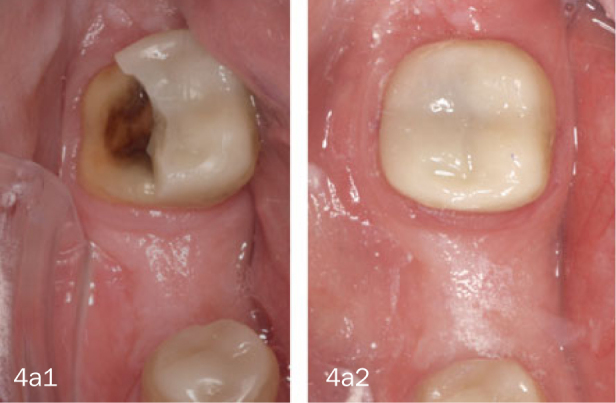
Adhesive fracture (1) and the repair (2) of restoration on tooth #47.

**Fig 4b:**
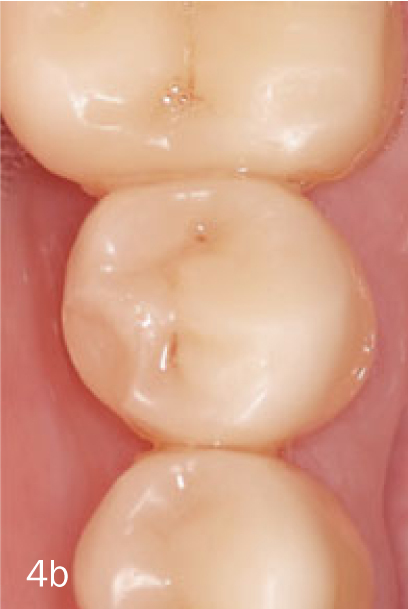
Chip frature of restoration on tooth #35.

Finally, the date of restoration placement, dates of both check-up visits, and failure type and reason were recorded. Patients completed both the OHIP-NL and OES-NL questionnaire at the recall appointment of 1 year.

### Statistical Analyses

Descriptive statistics were analyzed for all restorations. Success rates were calculated separately for indirect restorations and facial direct veneers for different failure levels. Furthermore, using descriptive statistics, the restorative status of abutment teeth (restored/endodontic treatment) was assessed as a possible risk factor for the failure of the restorative treatment.

Data from the questionnaires at baseline were compared with data after 1 year. The differences between the outcomes measured by questionnaires at the baseline and year 1 were analyzed using paired t-tests. This was feasible, as these differences were normally distributed. Mean scores for the OHIP-NL questionnaire were calculated and compared between timepoints, as were summary scores (questions no. 1 to 7) for OES-NL questionnaires. A separate analysis was completed for question no. 8 (overall impression question).

For analyzing the association of restoration failure (F1+ F2+F3) with tooth wear etiology, multilevel logistic regression analyses with a random intercept for patient were used. Separate analyses were completed for features of mechanical and chemical tooth wear lesions. The analyses were conducted within the R-software (v 3.6.2) and SPSS software (v 25). The significance level for all tests was set at p < 0.05.

## Results

### Patient Characteristics

**Fig 5:**
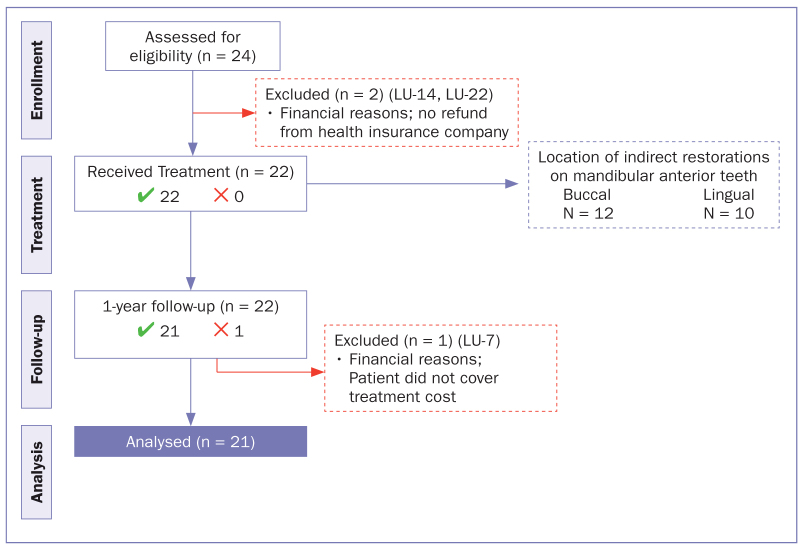
Flowchart of patients regarding allotted treatment, follow-up, and analysis.

Patients were recruited between February 2014 and January 2018. Twenty-two patients were treated using the treatment protocol, of whom 1 patient was lost to follow-up because of uncovered treatment costs (recall rate = 95%). Complete clinical follow-up was conducted for 21 patients (18 male; 3 female) with a mean age of 41.7 ± 10.4 years. The mean observation time was 13.5 ± 1.2 months (Fig 5).

### Clinical Data of Restorations

In total, 768 restorations were placed in 21 patients, comprising 200 direct facial veneer restorations and 568 indirect restorations. Of these indirect restorations, 158 were placed on molars, 163 on premolars, and 247 on anterior teeth. In 10 cases, mandibular anterior teeth received indirect lingual veneer restorations. In 12 cases, mandibular anterior teeth received indirect facial veneer restorations. The mean increment of VDO measured at the first molars was 2.8 ± 0.7 mm (Table 1).

**Table 1:** Overview of patient characteristics, treatment specifics, and clinical outcome

Patient characteristics											Treatment specifics				Clinical outcomes							
															F1: Replaced or lost		F2: Repaired		F3: Refurbished		All	
Patient number	Patient ID	Sex	Age (y)	Similar degree of wear for all occluding sextants	Imprint of mandibular anterior teeth in maxillary anterior teeth	Loss of convexities on the palatal surface of maxillary teeth	Presence of ‘raised restorations’	Enemal ‘cuff’ in gingival crevice of maxillary anterior teeth	# features of mechanical wear	# features of chemical wear	# Treated teeth	VDO increment at location of first molar (mm)	Night guard	Observation time (m)	Filtek Supreme XTE (n = 200)	LAVA Ultimate (n = 586)	Filtek Supreme XTE (n = 200)	LAVA Ultimate (n = 586)	Filtek Supreme XTE (n = 200)	LAVA Ultimate (n = 586)	All interventions	# interventions related to fracture
1	LU-1	m	66.3	1	1	0	0	0	2	0	25	2.9	yes	12.8	1					1	2	2
2	LU-2	m	39.9	0	0	1	1	1	0	3	26	1.7	yes	14.7								
3	LU-3	m	39.4	1	1	0	0	0	2	0	27	2.3	yes	13.5				3		5	8	8
4	LU-4	m	47.4	0	1	0	1	1	1	2	27	3.0	no	13.1								
5	LU-5	m	45.4	1	1	0	1	0	2	1	28	3.0	no	12.1			1	1		1	3	3
6	LU-6	m	51.6	1	0	1	1	1	1	3	28	3.2	no	13.2				1			1	1
7	LU-8	m	29.0	0	1	0	1	0	1	1	28	2.8	yes	14.8			2	1			3	3
8	LU-9	m	39.1	1	1	0	1	0	2	1	28	2.5	no	14.3			1	1			2	2
9	LU-10	f	42.5	0	1	0	1	0	1	1	28	3.3	no	12.9				1	2	2	5	5
10	LU-11	m	46.7	0	0	1	1	0	0	2	27	3.5	no	11.4								
11	LU-12	m	22.5	0	1	1	1	1	1	3	28	1.2	no	10.4				1			1	1
12	LU-13	m	31.0	0	1	0	0	0	1	0	28	3.0	no	14.0	1		2				3	2
13	LU-15	f	28.6	0	1	0	1	1	1	2	28	3.5	no	14.0								
14	LU-16	m	37.1	0	1	1	1	0	1	2	27	4.1	no	14.5								
15	LU-17	f	30.6	1	1	0	1	0	2	1	28	1.9	no	12.8				1			1	1
16	LU-18	m	30.3	1	0	1	1	0	1	2	28	2.9	no	15.8				1			1	1
17	LU-19	m	39.7	0	0	1	0	1	0	2	28	2.9	no	14.0								
18	LU-20	m	41.7	1	0	1	1	1	1	3	27	2.0	no	13.3								
19	LU-21	m	40.5	0	0	1	0	1	0	2	26	2.8	no	12.6								
20	LU-23	m	55.4	0	1	1	1	1	1	3	25	3.3	no	14.5				1			1	1
21	LU-24	m	24.9	1	0	1	1	1	1	3	28	3.4	no	13.8						1	1	1
Mean			41.7								27	2.8		13.5	2	0	6	12	2	10	32	31
SD			10.4								1	0.7		1.2								
Total																						

During the observation time, 32 interventions were carried out, of which 22 were performed on indirect restorations and 10 on facial veneers. The main reason for intervention was fracture (31 out of 32). No indirect restorations were replaced or lost. Two indirect restorations debonded and could be re-cemented, and were therefore regarded as a second-level failure (F2). Two facial veneers were replaced (Table 2). Success rates (%) of indirect restorations were calculated. Depending on the 3 failure levels, success rates were 100% (F1), 99.2% (F1+F2), and 97.2% (F1+F2+F3). Success rates of direct veneer restorations were 98.3% (F1), 97.9% (F1+F2), and 96.5% (F1+F2+F3) (Table 3).

**Table 2:** Overview of interventions

	Interventions			
		F1: Replaced	F2: Repaired	F3: Refurbished
Indirect restorations (n = 568)	Complete debonding		2	
	Adhesive fracture		10	
	Chip fracture			10
	TOTAL	0	12	10
Direct restorations (n = 200)	Bulk fracture	1		
	Adhesive fracture		6*	
	Chip fracture			2
	Esthetics*	1*		
	TOTAL	2	6	2
Total		2	18	12

*Restoration was replaced after a repair due to fracture, resulting in a poor esthetic outcome.

**Table 3:** Success rates

LAVA Ultimate restorations	Failure criteria	Group	No. of failures	Success at 1-year recall (%)
			
n = 568	F1	Whole group	0	100
	F1 + F2	Whole group	12	97.9
	F1+ F2 + F3	Whole group	22	96.1
Filtek Supreme XTE restorations				
n = 200	F1	Whole group	2	99.0
	F1 + F2	Whole group	7	96.5
	F1+ F2 + F3	Whole group	9	95.5
Subgroup analyses of LAVA Ultimate restorations per location				
n = 158	F1	Molar	0	100
	F1 + F2	Molar	7	95.6
	F1+ F2 + F3	Molar	13	91.8

n = 163	F1	Premolar	0	100
	F1 + F2	Premolar	4	97.5
	F1+ F2 + F3	Premolar	7	95.7

n = 247	F1	Incisors and canines	0	100
	F1 + F2	Incisors and canines	1	99.6
	F1+ F2 + F3	Incisors and canines	2	99.2

Of the 568 restored teeth, 10 had been endodontically treated at baseline. One indirect restoration was repaired. 148 restorations were placed on an abutment tooth containing a pre-existing composite restoration on the adhesive surface. This subgroup of restorations consisted of 10 veneers with no interventions and 138 indirect restorations with 11 interventions (out of 32) (see Supplement).

### Oral Health-related Quality of Life

Twenty patients completed the OHIP-NL questionnaire at baseline and after 1 year. After 1 year, the treatment resulted in a significant improvement of the OHRQoL, as the questionnaire of the OHIIP-NL had a statistically significantly lower mean score. A mean difference per question of –0.7 ± 0.5 (p < 0.001) was found. Nineteen patients completed the OES-NL questionnaires at baseline and after 1 year. The esthetic appearance also significantly improved: for both the summary score of questions no. 1 to 7 (average difference of 29.6 [p < 0.001]) and the overall impression score (average change 3.7 [p < 0.001]), a significantly positive change was found. The changes in OHRQoL are presented in Table 4.

**Table 4:** OHRQoL analyses: comparison (paired t-test) of baseline and 1-year OHIP mean score (± SD), OES summary score (± SD), and OES overall impression score (± SD)

	n	Mean baseline	Mean after 1 year	Mean difference	95% CI of difference	p-value
OHIP mean score	20	2.0 (0.6)	1.3 (0.2)	0.7 (0.5)	0.4 – 1.0	< 0.001
OES summary scores No. 1 to 7	19	29.7 (9.9)	59.3 (5.2)	29.6 (12.9)	24.4 – 35.8	< 0.001
OES No. 8 (overall impression)	19	4.8 (1.6)	8.5 (1.0)	3.7 (0.5)	2.7 – 4.7	< 0.001

### Association with Wear Etiology Features

A first multilevel logistic regression analysis was conducted to determine whether the presence of features of mechanical tooth wear at baseline was associated with a higher risk of restoration failure (Table 5). The presence of 1 or 2 features showed no significantly increased risk of restoration failure compared to the absence of these features (p = 0.78; OR=7.4e7; 95%CI=1.33–65.46). The analysis was repeated for the relation between the presence of features of chemical tooth wear and restoration failure. The presence of 2 or 3 features, compared to the absence of these features, resulted in a significantly lower risk of failure, with a p-value of 0.002 (OR: 0.03; 95%CI: 0.002–0.197) and 0.004 (OR: 0.15; 95%CI: 0.037–0.539), respectively.

**Table 5:** Multilevel logistic regression analyses for risk assessment of tooth wear phenotypes

All restorations in prospective study (n = 756)		N patients (total: n = 21)	Interventions (F1+F2+F3) (total: n = 31)	OR	p-value	95% CI
Number of features of mechanical tooth wear at baseline	None (reference)	4	0	n.a.	–	–
	1	12	15	2.8 e+7	0.79	0.547–22.54
	2	5	16	7.4 e+7	0.78	1.33–65.46
Number of features of chemical tooth wear at baseline	None (reference)	3	12	n.a.	–	–
	1	5	14	0.61	0.32	0.212–1.84
	2	7	1	0.03	0.002	0.002–0.197
	3	6	4	0.15	0.004	0.037–0.539

## Discussion

The aim of this study was to evaluate the clinical performance of minimally invasive, CAD/CAM nano-ceramic composite restorations in patients with severe tooth wear. An additional aim was to evaluate the effect of the restorative treatment on OHRQoL and evaluate the etiology of tooth wear as a risk factor for restoration failure. In the short term, success rates between 100% and 97.2% were found, with fracture as the main failure modality. This is comparable to an established treatment option for tooth wear: directly applied composite restorations.^3,11,19,20,24,31^ Furthermore, OHRQoL improved significantly. Such outcomes are not frequently reported related to the clinical performance of restorations, but should be considered more often in the context of patient-centered care. Although the follow-up of this trial was short, it can be assumed that for the restorative treatment of tooth wear, minimally invasive, indirect CAD/CAM restorations without retentive geometry were successful after 1 year.

Reporting about small material chippings as an outcome for the survival of restorations may be useful to demonstrate a tendency in studies with a limited observation time. Susceptibility to chipping can be material- and technique-specific and might have predictive value for clinical behavior in the longer term. In addition, reporting about these outcomes is useful for optimal comparison with other studies. However, from the perspective of good care, refurbished restorations are clinically acceptable and therefore arguably successful.

Despite the presence of bruxism and the non-retentive geometry of restorations, only 2 complete debondings occurred out of 568 tabletop LAVA Ultimate restorations, confirming its good adhesive ability. These findings are not congruent with the findings of a prospective study evaluating 50 LAVA Ultimate single crowns on implant-borne zirconia abutments with a retentive geometry; the survival rate was only 14% after 1 year^29^ after multiple fractures and multiple debondings occurred. That study resulted in the manufacturer’s retraction of the indication for LAVA Ultimate as a single-crown material. As adhesive procedures were almost identical, ie, air abrasion, silane application, and the resin cement were identical, it seems likely that the substrate greatly influences the survival rate of the LAVA Ultimate restorations. This is supported by findings of another study^18^ that identified failure reasons for these particular single crowns on zirconia abutments, claiming the weakest link to be the adhesive layer between composite cement and zirconia, and not the composite-cement/LAVA-Ultimate interface. To assess the influence of the substrate on the survival rate of LAVA Ultimate restorations in this study, abutment teeth were checked for their restorative status; the presence of a composite restoration, that was sandblasted and treated with a silane in the present study, could be a risk factor for clinical failure. However, no signs or indications of a higher incidence of failure on restored teeth were found, at least not within the short observation time.

Patients suffering from severe tooth wear can be considered as high-risk patients with a destructive oral environment that is likely to influence the longevity of restorations. While most patients only showed 0 or 1 interventions, 5 patients (patients 3, 5, 8, 10, and 13) received 69% of all interventions (22 of 32) (see Supplement). Tooth wear is a multifactorial phenomenon involving both chemical and mechanical stress. We speculated that bruxism, related to mechanical stress, would be a risk factor for the survival of restorations,^35^ as frequent high masticatory forces may lead to early fracture of restorations. Meanwhile, dental restorations potentially protect tooth tissues fromr chemical wear; we therefore speculated that patients suffering from chemical wear would demonstrate superior longevity of restorations. To substantiate the operators’ initial supposition about the the etiology of patients’ tooth wear, morphological features of tooth wear at baseline were scored. Five features were selected, of which 4 showed a significantly higher prevalence of either chemical (3 features) or mechanical (1 feature) tooth wear in a previous study.^12^ An additional feature of mechanical tooth wear was scored – the imprint of mandibular anterior teeth in palatal surfaces of maxillary anterior teeth – as it can only be explained by mechanical stress.

Surprisingly, the presence of features of mechanical wear did not show a significantly higher odds ratio (OR) compared to the absence of these features. However, the presence of features of chemical wear, compared to absence, showed a statistically significant lower OR, indicating a lower risk of failure when features of chemical tooth wear were present. This might be explained by the fact that features of mechanical tooth wear are detectable in two situations: 1) patients with high mechanical stress and 2) patients with moderate mechanical stress in combination with chemical stress. In the latter situation, chemical stress leads to softening of hard tooth tissue, making it susceptible to tooth wear at low(er) mechanical stress.^4,9^

## Conclusion

In patients with severe tooth wear, features of chemical tooth wear may be discriminatory for distinguishing between cases of high mechanical stress (in the absence of features of chemical tooth wear) and cases of mild to moderate mechanical stress (in the presence of features of chemical tooth wear). We therefore consider the absence of features of chemical wear as a risk factor for restoration survival.

## Supplement

**Table:** 

Patient Characteristics																								
							Restorative Treatment																	
							Overall			Filtek Supreme XTE			Lava Ultimate											
							All restorations			Buccal veneer			Anterior			Premolars			Molars			Overall		
Patient ID	Sex	Age (y)	Observation time (m)	# Treated Teeth	VDO increment (mm)	Night guard	# Restorations	# pre-existing restoration in adhesive suface	# endodontically treated teeth at baseline	# Filtek Supreme restorations	# pre-existing restoration in adhesive suface	# endodontically treated teeth at baseline	# Lava Ultimate anterior restorations	# pre-existing restoration in adhesive suface	# endodontically treated teeth at baseline	# Lava Ultimate anterior restorations	# pre-existing restoration in adhesive suface	# endodontically treated teeth at baseline	# Lava Ultimate anterior restorations	# pre-existing restoration in adhesive suface	# endodontically treated teeth at baseline	# Lava Ultimate anterior restorations	# pre-existing restoration in adhesive suface	# endodontically treated teeth at baseline
LU-1	m	66,3	12,8	25	2,9	Yes	40	21	0	15	4	0	11	5	0	8	6	0	6	6	0	25	17	0
LU-2	m	39,9	14,7	26	1,7	Yes	34	3	0	12	0	0	8	0	0	6	0	0	8	3	0	22	3	0
LU-3	m	39,4	13,5	27	2,3	Yes	33	7	0	6	0	0	12	0	0	8	1	0	7	6	0	27	7	0
LU-4	m	47,4	13,1	27	3,0	No	34	8	0	7	0	0	12	2	0	8	2	0	7	4	0	27	8	0
LU-5	m	45,4	12,1	28	3,0	No	40	9	0	12	3	0	12	2	0	8	2	0	8	2	0	28	6	0
LU-6	m	51,6	13,2	28	3,2	No	36	10	2	8	1	2	12	3	2	8	0	0	8	6	0	28	9	2
LU-8	m	29,0	14,8	28	2,8	Yes	40	5	1	12	0	1	12	1	1	8	1	0	8	3	0	28	5	1
LU-9	m	39,1	14,3	28	2,5	No	40	4	0	12	2	0	12	0	0	8	0	0	8	2	0	28	2	0
LU-10	v	42,5	12,9	28	3,3	No	40	12	0	12	0	0	12	0	0	8	4	0	8	8	0	28	12	0
LU-11	m	46,7	11,4	27	3,5	No	37	12	3	10	0	0	12	0	0	8	5	1	7	7	2	27	12	3
LU-12	m	22,5	10,4	28	1,2	No	40	0	0	12	0	0	12	0	0	8	0	0	8	0	0	28	0	0
LU-13	m	31,0	14,0	28	3,0	No	38	1	0	10	0	0	12	0	0	8	1	0	8	0	0	28	1	0
LU-15	v	28,6	14,0	28	3,5	No	40	3	0	12	0	0	12	0	0	8	0	0	8	3	0	28	3	0
LU-16	m	37,1	14,5	27	4,1	No	32	8	0	6	0	0	12	0	0	8	2	0	6	6	0	26	8	0
LU-17	v	30,6	12,8	28	1,9	No	42	2	2	14	0	0	12	0	0	8	0	0	8	2	2	28	2	2
LU-18	m	30,3	15,8	28	2,9	No	28	11	0	0	0	0	12	0	0	8	4	0	8	7	0	28	11	0
LU-19	m	39,7	14,0	28	2,9	No	36	2	0	8	0	0	12	0	0	8	0	0	8	2	0	28	2	0
LU-20	m	41,7	13,3	27	2,0	No	35	12	0	8	0	0	12	2	0	8	3	0	7	7	0	27	12	0
LU-21	m	40,5	12,6	26	2.8	No	32	5	1	6	0	0	12	0	0	6	0	0	8	5	1	26	5	1
LU-23	m	55,4	14,5	25	3,3	No	37	7	0	12	0	0	12	0	0	7	4	0	6	3	0	25	7	0
LU-24	m	24,9	13,8	28	3,4	No	34	6	1	6	0	1	12	1	1	8	1	0	8	4	0	28	6	1
Mean		41,7	13,5	27	2,8		37	7	0	10	0	0	12	1	0	8	2	0	8	4	0	27	7	0
SD		10,4	1,2	1	0,7		4	5	1	3	1	0	1	1	0	1	2	0	1	2	1	1	4	1
Total							768	148	10	200	10	4	247	16	4	163	36	1	158	86	5	568	138	10

**Table:** 

	Clinical Observations																		
	Failures																		
	Filtek Supreme XTE				Lava Ultimate												Total		
	Buccal veneers				Anterior teeth (I+C)				Premolar				Molar						
	F1	F2	F3	Compromized substrate	F1	F2	F3	Compromized substrate	F1	F2	F3	Compromized substrate	F1	F2	F3	Compromized substrate	Failures	On compromised substrate	On endodontically treated teeth
1			0							1	1					2	1	
																0		
									1	1	1		2	4	5	8	6	
																0		
	1		0										1	1	1	3	1	
													1		0	1		
	2		0										1		0	3		
	1		0		1		0									2		
		2	0			1	0		1	1	2					5	2	
																0		
									1		0					1		
1	2		0													3		
																0		
																0		
													1		1	1	1	1
													1		1	1	1	
																0		
																0		
																0		
									1		0					1		
														1	0	1		
Total	2	6	2	0	0	1	1	0	0	4	3	4	0	7	6	8	32	12	1
F1	2				0				0				0				2		
F2		6				1				4				7			18		
F3			2				1				3				6		12		

## Clinical relevance:

The use of minimally invasive, CAD/CAM nano-ceramic composite restorations in the restorative treatment of severely worn dentitions showed satisfactory results in the short term.
